# Thermomechanical data of polyurethane shape memory polymer: Considering varying compositions

**DOI:** 10.1016/j.dib.2020.106294

**Published:** 2020-09-09

**Authors:** Hailey Fisher, Payton Woolard, Colton Ross, Robert Kunkel, Bradley N. Bohnstedt, Yingtao Liu, Chung-Hao Lee

**Affiliations:** a Biomechanics and Biomaterials Design Laboratory (BBDL), School of Aerospace and Mechanical Engineering, The University of Oklahoma, Norman, OK 73019, USA; b Department of Neurological Surgery, Indiana University School of Medicine, Indianapolis, IN 46202, USA; c Smart Materials and Intelligent Systems (SMIS) Laboratory, The University of Oklahoma, Norman, OK 73019, USA; d Institute of Biomedical Engineering, Science and Technology (IBEST), The University of Oklahoma, Norman, OK 73019, USA

**Keywords:** Shape memory polymer, Thermomechanical properties, Glass transition temperature, Thermal degradation threshold, Failure stress, Failure strain

## Abstract

This article presents data from the investigation of the thermal characteristics and mechanical behaviors of twelve different compositions of a polyurethane shape memory polymer (SMP). Each of the SMP compositions has a unique molar ratio of three monomers: (i) hexamethylene diisocyanate (HDI), (ii) N,N,N′,N′-Tetrakis(2-Hydroxypropyl)ethylenediamine (HPED), and (iii) Triethanolamine (TEA). The thermal characteristic datasets for each composition include the glass transition temperatures, as obtained from differential scanning calorimetry (DSC) and dynamic mechanical analysis (DMA), and the thermal degradation thresholds, as found from thermogravimetric analysis (TGA). The mechanical behaviors of the SMPs are represented by the failure stresses and strains, as obtained by cyclic tensile testing and failure testing, respectively. The interpretation of these measurements as well as a discussion of the potential usage of candidate SMP compositions for medical devices can be found in the companion article by Kunkel *et al*. (2018) [1], “Synthesis and characterization of bio-compatible shape memory polymers with potential applications to endovascular embolization of intracranial aneurysms.”

## Abstract (author highlights)

Value of the Data

• The data presented in this DiB article will provide quantitative measurements of the thermal characteristics and mechanical properties of twelve different compositions of aliphatic polyurethane shape memory polymers (SMPs), associated with glass transition temperatures that may permit their usage in biological applications.
• This information can assist researchers in making informed selections or comparisons of SMP compositions for research applications, such as SMP-based endovascular embolic aneurysm devices or other biomedical devices.
• The glass transition temperatures and elastic modulus reduction values may be used for in the development of an SMP-based device with desired biologically compatible actuation temperature and mechanical properties for endovascular aneurysm occlusion.
• This data may also be useful in informing researchers of adjustments to be made to the SMP molar compositions that will allow fine tuning for targeted SMP thermomechanical properties for specific applications, as performed by Landsman *et al.* (2016) in the development of an SMP-based peripheral occlusion device [2].

## Specifications Table

**Table:** 

Subject	Polymers and Plastics
Specific subject area	Thermal and mechanical properties of polyurethane shape memory polymer
Type of data	Table Figure .txt Text Files .xlsx Excel Files .m MATLAB Script File for Plotting the Data
How data were acquired	Instruments used for thermomechanical characterizations: (i) dynamic mechanical analysis (TA Instruments, Q800) (ii) differential scanning calorimetry (TA Instruments, Q20) (iii) thermogravimetric analysis (TA Instruments, Q50) (iv) uniaxial tensile testing (Instron, 5969)
Data format	(i) Raw (ii) Analyzed (data may be further analyzed using the included SMP_plots_calculations.m MATLAB script)
Parameters for data collection	SMPs of twelve varying ratios of HDI, HDEP, and TEA were considered: HDI: 53.5-62.3% of the total composition HPED: 2.7-46.5% of the total composition TEA: 0.0-35.0% of the total composition All the specimens were fabricated in a nitrogen-protected environment.
Description of data collection	(i) TA instrument Q800 in tension mode was used for DMA testing. (ii) TGA was performed using a TA Q50. (iii) DSC was done using a TA Q20. (iv) All mechanical testing was performed under an Instron uniaxial mechanical testing machine with a thermal-control chamber. (iii) An in-house MATLAB (MathWorks) program was used to determine the onset temperature of thermal degradation, which was used as reference for the DSC measurements.
Data source location	School of Aerospace and Mechanical Engineering, The University of Oklahoma, Norman, OK 73019, USA (35°12’36.6”N, -97°26’35.3”W)
Data accessibility	With the article
Related research article	Kunkel, R.P., Laurence, D.W., Wang, J., Robinson, D., Scherrer, J., Wu, Y., Bohnstedt, B.N., Chien, A., Liu, Y., and Lee, C. H., 2018, "Synthesis and characterization of bio-compatible shape memory polymers with potential applications to endovascular embolization of intracranial aneurysms," J Mech Behav Biomed Mater, 88, pp. 422-430. https://doi.org/10.1016/j.jmbbm.2018.08.037

## 1 Data Description

The included data concerns the thermomechanical and tensile properties of twelve different SMP compositions with varied molar ratios of the monomers hexamethylene diisocyanate (HDI), N,N,N′,N′-tetrakis(2-hydroxypropyl) ethylenediamine (HPED), and triethanolamine (TEA). The molar ratios used in each composition, labelled SMP1-12, are found in Table 1 from the companion article [1]. The thermomechanical properties of each composition were examined by dynamic mechanical analysis (DMA), dynamic scanning calorimetry (DSC), and thermogravimetric analysis (TGA). Uniaxial cyclic tensile testing and failure testing were used to obtain the mechanical properties for each composition. A summary of the contents of the included data files can be found in the Appendix.

**Table 1:** The elastic moduli from uniaxial cyclic tensile testing and the percent reduction in elastic moduli with respect to the first cycle for each SMP composition. The elastic modulus values (in MPa) for the first cycle are provided in the first row. Data are presented as mean±standard error of the mean (n = 2).

SMP Composition	Cycle 1 Elastic Modulus (MPa)	Cyclic Elastic Modulus Reduction with respect to Cycle 1 (%)								
		Cycle 2	Cycle 3	Cycle 4	Cycle 5	Cycle 6	Cycle 7	Cycle 8	Cycle 9	Cycle 10
SMP1	24.35 ± 0.10	3.97 ± 0.77	4.68 ± 0.88	5.23 ± 0.85	5.27 ± 1.02	5.36 ± 1.07	5.42 ± 1.01	5.60 ± 0.96	5.68 ± 0.71	7.24 ± 0.73
SMP2	21.34 ± 0.29	2.04 ± 0.36	2.34 ± 0.44	2.61 ± 0.80	2.80 ± 0.81	2.79 ± 0.89	2.78 ± 0.83	2.99 ± 1.17	2.76 ± 1.01	2.83 ± 1.12
SMP3	19.02 ± 0.29	0.61 ± 0.48	1.16 ± 0.13	1.15 ± 0.00	1.20 ± 0.06	0.84 ± 0.10	0.77 ± 0.22	-0.26 ± 0.94	0.24 ± 0.38	0.26 ± 0.25
SMP4	20.44 ± 0.04	1.87±0.53	2.50 ± 0.46	2.60 ± 0.50	2.69 ± 0.55	2.88 ± 0.45	2.87 ± 0.43	2.82 ± 0.48	2.92 ± 0.49	2.94 ± 0.43
SMP5	19.92 ± 0.96	2.03 ± 0.46	2.57 ± 0.63	2.82±0.77	2.91 ± 0.73	2.94 ± 0.75	2.96 ± 0.85	3.01 ± 0.86	2.91±0.75	3.03 ± 0.85
SMP6	21.02 ± 1.58	1.91 ± 0.08	2.49 ± 0.20	2.86 ± 0.10	2.91±0.30	3.03 ± 0.15	3.06 ± 0.17	3.05±0.31	3.02 ± 0.27	2.98 ± 0.27
SMP7	18.81 ± 0.06	1.25 ± 0.09	1.67 ± 0.19	1.71 ± 0.07	1.87 ± 0.32	1.84 ± 0.50	1.82 ± 0.50	1.78 ± 0.46	1.68 ± 0.48	1.68 ± 0.68
SMP8	19.45 0.90	1.31 ± 0.34	1.75 ± 0.27	2.01 ± 0.51	2.14 ± 0.70	2.05 ± 0.67	2.11 ± 0.62	2.01 ± 0.64	1.88 ± 0.56	2.06 ± 0.54
SMP9	18.50 2.09	0.43 ± 0.45	0.98 ± 0.56	0.85 ± 0.49	0.95 ± 0.54	0.82 ± 0.40	1.01 ± 0.50	1.07 ± 0.46	0.85 ± 0.29	0.87 ± 0.26
SMP10	15.87 0.98	0.75 ± 0.20	-0.47 ± 1.25	-2.35 ± 3.36	0.31 ± 0.60	0.57 ± 0.72	0.59 ± 0.65	0.60 ± 0.64	0.49 ± 0.61	0.49 ± 0.45
SMP11	15.48 0.26	0.74 ± 0.00	0.80 ± 0.21	0.95 ± 0.05	1.19 ± 0.08	1.21 ± 0.18	1.09 ± 0.05	1.35 ± 0.37	1.46 ± 0.13	1.40 ± 0.05
SMP12	13.37 0.39	1.30 ± 0.27	1.24 ± 0.12	1.28 ± 0.46	1.59 ± 0.47	1.62 ± 0.44	1.38 ± 0.66	1.31 ± 0.41	1.67 ± 0.39	1.68 ± 0.53

**Fig. 1:**
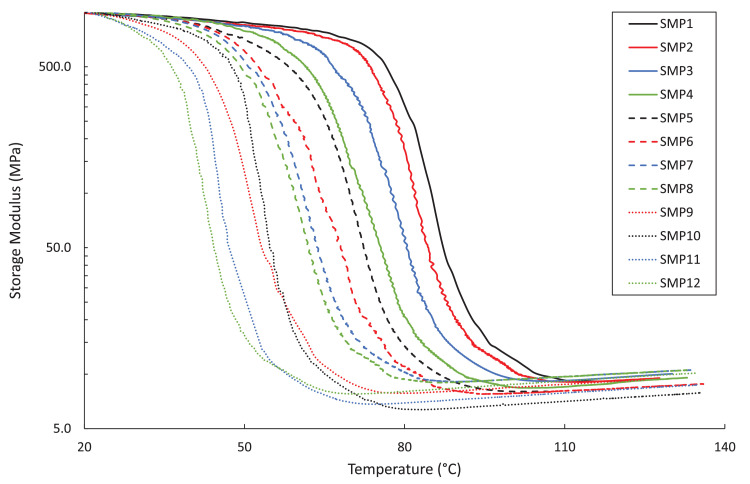
Storage moduli (MPa) for all twelve SMP compositions as a function of temperature (°C), as found through the DMA testing.

**Fig. 2:**
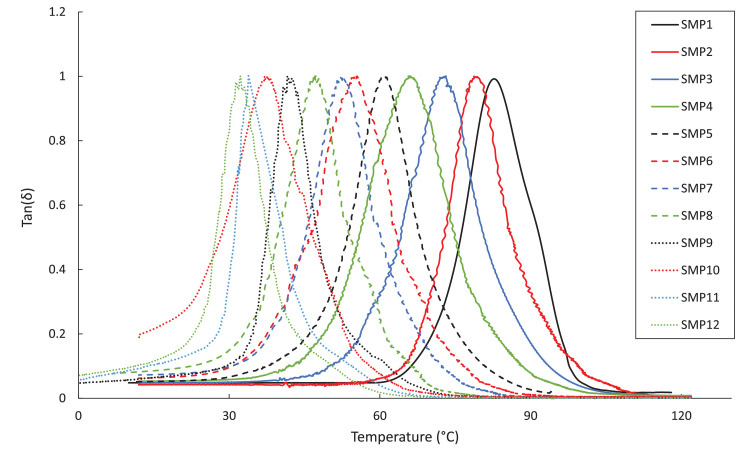
DMA testing results of the tan(δ) values for each SMP composition versus temperature (°C).

**Fig. 3:**
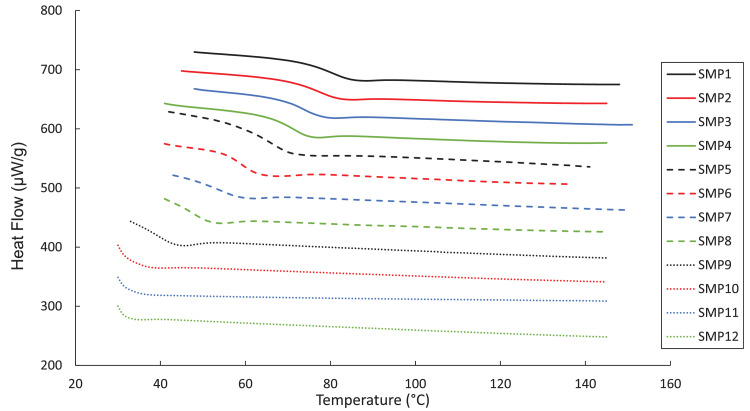
DSC testing results of the heat flow (μW/g) versus temperature (°C).

**Fig. 4:**
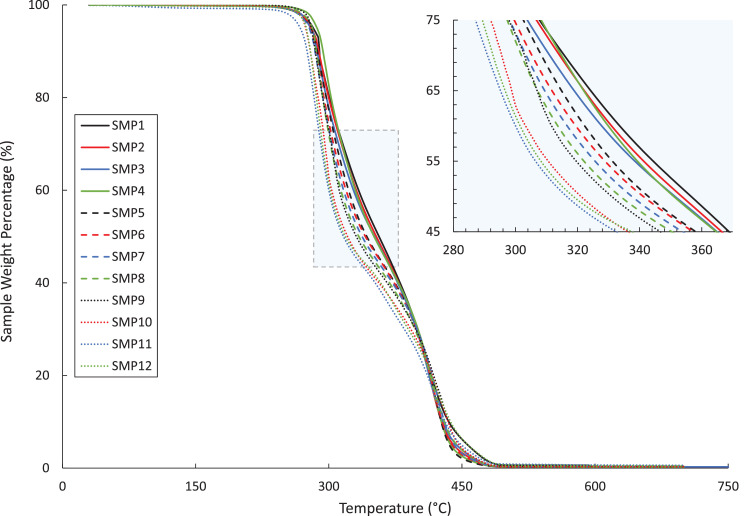
Weight percentage of the remaining SMP versus temperature (°C), obtained via the TGA for the twelve SMP compositions.

The DMA, DSC, and TGA measurements are included in four .txt files. Each file contains three columns – Column 1: the composition label (1–12), Column 2: the testing environment temperature in degree Celsius, and Column 3: the test-specific physical quantity. The test-specific physical quantity provided in the third column of each .txt file is defined as follows: “DMA_storage.txt”, the shear storage moduli (MPa) (Fig. 1); “DMA_tanDelta.txt”, the tan(δ) values (Fig. 2); “DSC_data.txt”, the heat flows (µW/g) from the second DSC testing cycle (Fig. 3); “TGA_data.txt”, the remaining weight percentages of the SMP sample (Fig. 4). From the data contained in each .txt file, essential thermal characteristics for each SMP composition may be derived, such as the glass transition temperature (T_g_), as shown in Table 2 in the companion article [1]. Specifically, T_g_ for a sample may be found from the DMA data as the temperature corresponding to the maximum tan(δ) value. Using the DSC .txt file, T_g_ is the temperature at which the local minimum of heat flow (μW/g) occurs.

**Fig. 5:**
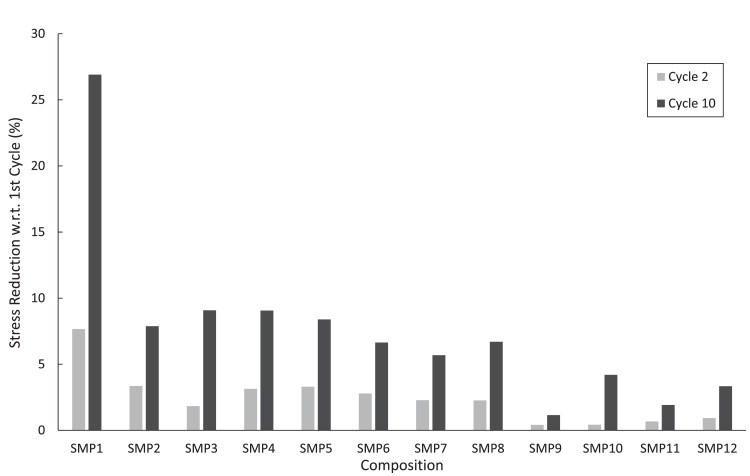
Average stress reduction values (%) relative to the first cyclic loading from cyclic tensile testing at the second and the tenth cycles, for each SMP composition.

**Fig. 6:**
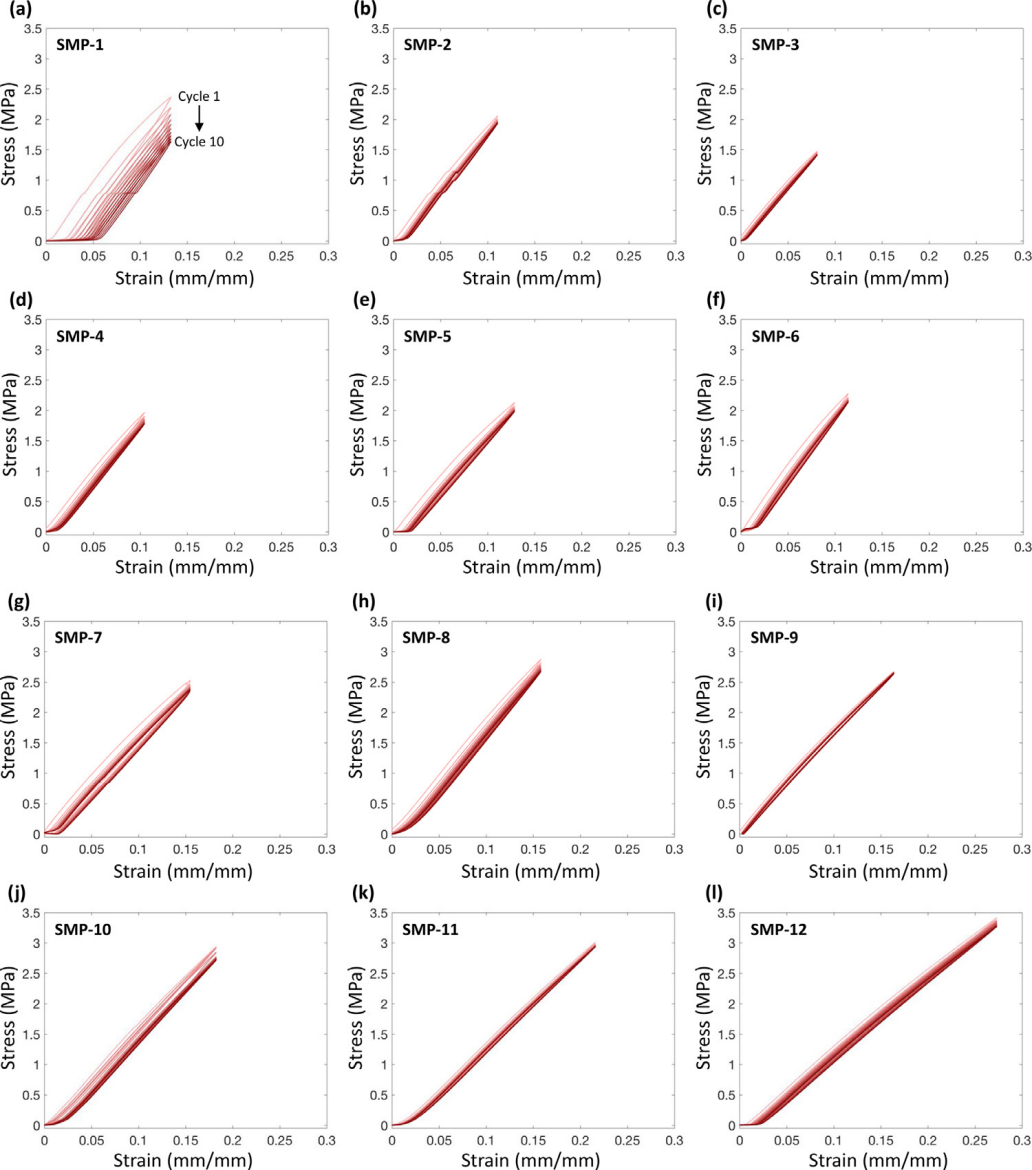
Measured stress and strain for ten cycles of loading of a representative dog-bone sample specimen of each SMP composition: (a) SMP-1, (b) SMP-2, (c) SMP-3, (d) SMP-4, (e) SMP-5, (f) SMP-6, (g) SMP-7, (h) SMP-8, (i) SMP-9, (j) SMP-10, (k) SMP-11, and (l) SMP-12. The red gradient of increasing darkness shows the progression from Cycle 1 to Cycle 10.

The mechanical data obtained from the uniaxial cyclic testing and failure testing are contained within the .xlsx workbooks: “Uniaxial_cyclic_tensile.xlsx” and “Failure_data.xlsx”, respectively. In each Excel workbook, there is one sheet dedicated to each tested specimen and multiple specimens included for each composition. The sheets are named in the format “SMP-X_Y”, where X denotes the composition number carrying a value of 1–12, and Y indicates the ID for the specific tested specimen. Within each sheet, the data is organized in columns (from the first to the last): (i) the testing time (s), (ii) the total cycle count, (iii) the extension (mm), (iv) the applied load (N), (v) the tensile stress (MPa), and (vi) the tensile strain (mm/mm). The average elastic modulus reduction relative to the first cycle for each SMP composition is shown in Table 1, whereas Fig. 5 shows the average stress reduction for the second and tenth cycles, relative to the first cycle, for each SMP composition. In addition, the stress-strain curves from the cyclic uniaxial testing of representative specimens of each SMP composition are found in Fig. 6.

## 2 Experimental Design, Materials and Methods

### 2.1 Shape memory polymer synthesis and sample preparation

For a detailed account of the SMP synthesis process, we refer the reader to Kunkel *et al*. [1] and Wilson *et al*. [3]. In brief, the synthesis of SMP follows a six step process: (i) secure a sealed nitrogenous environment; (ii) measure the desired molar masses of HPED, and TEA into a glass beaker; (iii) measure the desired mass of HDI into a separate disposable plastic container; (iv) use a stirring device to mix the monomers in the glass beaker; (v) add the HDI from the plastic container to the HPED and TEA in the glass beaker and stir slowly; and (vi) stop stirring the mixture and remove the SMP from the nitrogen environment when the liquid becomes transparent.

### 2.2 Quantification of the thermomechanical properties of the synthesized SMPs

DMA, DSC, and TGA were used to quantify the thermomechanical properties of each of the twelve SMP compositions. DMA was used to find the storage modulus, loss modulus, and glass transition temperatures (T_g_). DSC heat flow plots were used for verification of the T_g_ value obtained from DMA. TGA was used to find the thermal stability of each SMP composition.

DMA (TA Instruments, Q800) was performed in a nitrogen environment on SMP beams measuring 45 mm x 8 mm x 1 mm. Samples were heated from 20°C to 120°C at a rate of 5°C/min in tension mode. The cyclic loading frequency was set to 1 Hz. From DMA, the storage modulus, loss modulus, and tan(δ) as a function of the temperature were retrieved for each specimen as a direct output of the testing device. The curves in Fig. 2 were creating by plotting the output tan(δ) versus the instantaneous temperature, which was collected from the device. The temperature corresponding to the maximum of the tan(δ) curve was determined as the T_g_, as recommended by the instrument's manufacturer [4].

The TA Q20 (TA Instruments) was used to perform a cyclic DSC process on samples in a nitrogenous environment. In each cycle, samples were heated from 20°C to 160°C at 5°C/min increments, cooled from 160°C to 20°C with 50°C/min increments, and held at 20°C for 3 min. Measurements were visualized on standard heat flow plots, and the glass transition temperature for each sample was determined as the local minimum of the heat flow as a function of temperature.

TGA (TA Instruments, Q50) was used to determine the thermal degradation threshold temperature. SMP samples were heated at 10°C/min from 31°C to 600°C in a nitrogen environment. The thermal stability of each sample was defined with an in-house MATLAB (MathWorks, MA) program that pinpointed the onset of thermal degradation as the intersection of two linear regressions between the TGA curves: below the T_g_ and between 90% and 85% of the remaining mass. For more details about performing linear regression, we refer the reader to Ref. [5].

### 2.3 Mechanical testing for the synthesized SMPs

Failure testing and cyclic tensile testing (Instron 5969) were used to characterize the mechanical behaviors of each SMP composition. Specifically, failure testing provided the failure stress and failure strain, while cyclic testing quantified the changes in the elastic modulus and the cyclic stress reductions.

For both mechanical tests, ASTMD638 dog-bone specimens [6] were created for each of the twelve SMP compositions. The dimensions of the testing region were measured along several locations to calculate the undeformed (original) cross-sectional area (*A*) of each specimen, which was later used in the calculation of the nominal stress,

(1) $$\sigma =\frac{F}{A}$$

where *F* is the instantaneous load applied by the Instron testing device. Cyclic stress reduction values reported in Figure 5 were calculated by

(2) $$SR_{i}=\left(1-\frac{\sigma_{\max,i}}{\sigma_{\max,1}}\right)\times 100\%,$$

where the subscript *i* indicates the cycle number and *σ*_max , *i*_ is the maximum stress of cycle *i*. Furthermore, the elastic modulus can be quantified as the slope of a linear portion of the unloading curve using the standard equation

(3) $$E=\frac{\sigma }{\varepsilon },$$

where ɛ is the normal strain (i.e. the change in the length of the specimen divided by the initial length, $\varepsilon =\delta /L_{0}$). The elastic modulus reduction with respect to the first cycle can then be found with

(4) $$ER_{i}=\left(1-\frac{E_{i}}{E_{1}}\right)\times 100\%,$$

where *E_i_* is the elastic modulus measured for the *i*th cycle. The included MATLAB script file may be used to derive these stress quantities from the Uniaxial_cyclic_tensile.xlsx data file.

To avoid slippage during testing, double-sided padded tape was added to both sides of the dog-bone specimen's gripping regions before mounting. The extension reading of the Instron mechanical testing machine was calibrated after each specimen was mounted and before testing began, according to the procedure described by Kunkel *et al.* [1].

Tensile failure testing and cyclic testing were performed at 10°C above the T_g_ of each specimen with a strain rate of 2 mm/min. Five failure tests were performed for each specimen, and the most consistent three tests (as determined by the elastic modulus and failure stress values) were utilized in the subsequent analyses. For cyclic testing, each sample underwent three cycles of preconditioning at 25% of the failure strain, before being subjected to ten loading and unloading cycles at 50% of the failure strain.

## Declaration of Competing Interest

The authors declare that they have no known competing financial interests or personal relationships which have, or could be perceived to have, influenced the work reported in this article.

## Figures and Tables

**Table:** 

Supplementary File Name	Description	Related Figure(s) or Table(s)
Uniaxial_cyclic_tensile.xlsx	Raw uniaxial cyclic tensile testing data	Table 1 Fig. 5, Fig. 6
Failure_data.xlsx	Raw cyclic failure testing data	
TGA_data.txt	Raw data from TGA testing	Fig. 4
DSC_data.txt	Raw data from DSC testing	Fig. 3 Table 2 in companion article [1]
DMA_tanDelta.txt	Raw data from DMA testing	Fig. 2 Table 2 in companion article [1]
DMA_Storage.txt	Raw data from DMA testing	Fig. 1
SMP_plots_calculations.m	Analyze and plot data from Uniaxial_cyclic_tensile.xlsx	

## Appendix B Supplementary materials

Image, application 1

Image, application 2

Image, application 3

Image, application 4

Image, application 5

Image, application 6

Image, application 7
